# Spatial Accessibility of Primary Health Care in Rural Areas in Poland

**DOI:** 10.3390/ijerph18179282

**Published:** 2021-09-02

**Authors:** Łukasz Lechowski, Angelika Jasion

**Affiliations:** Faculty of Geographical Sciences, University of Lodz, Kopcińskiego 31, 90-142 Łódź, Poland; lukasz.lechowski@geo.uni.lodz.pl

**Keywords:** spatial accessibility, primary health care, rural areas, spatial autocorrelation, GIS

## Abstract

The aim of the study was to assess the spatial accessibility of basic and universal healthcare (understood as primary healthcare (PHC) facilities) in rural statistical localities in Poland. Data from the National Health Fund, Central Statistical Office, National Register of Geographic Names and OpenStreetMap were used in the research. The research was carried out on the basis of modelled distance from the rural statistical localities to the nearest PHC facility. The methods used included network analysis, characteristics of normal point distribution, Theil index, and spatial autocorrelation. Areas where the greatest shortages of access to PHC facilities occurred were indicated on the basis of the analysis of their clustering density. The average distance from rural statistical localities in Poland to PHC facilities is about 5 km. Slightly more than 70% of the distance values are within one standard deviation of the mean. Better access to the examined healthcare facilities is available in the southern and central parts of Poland, while northern and eastern Poland, as well as the border areas, suffer from lower accessibility. Poor access to PHC occurs first of all at the border of Greater Poland Voivodeship with the Kuyavian–Pomeranian Voivodeship, on the border of the Lodz Voivodeship, in Masovian and Swietokrzyskie Voivodeship, and in the ring surrounding Warsaw, as well as in the Pomeranian Voivodeship. The research findings can be used to develop strategies to improve the accessibility of primary care facilities in rural areas.

## 1. Introduction

In Poland, according to GUS data, one in five households in rural areas does not have access to a car. At the same time, rural areas are usually characterized by poor access to public transport [1,2]. In view of the limitations resulting from the mobility of the population, equal access of citizens to basic healthcare, which is a fundamental obligation of the State guaranteed in the Constitution of the Republic of Poland, becomes a significant problem. This problem concerns many countries, and the availability of healthcare in rural areas depends on the distance between rural areas and the main centres providing medical services, adequate diagnostic equipment at local facilities, employing qualified medical staff, the number of pharmacies, and the possibility of taking advantage of specialist health and prevention services [3,4,5]. This problem is all the more important as access to good PHC can support an infectious disease vaccination program such as that for COVID-19. It allows for better access to vaccination information from a physician who is familiar with the patient’s condition. In turn, the PHCs themselves can be the site of vaccine distribution at the local level. Good access to such services also significantly reduces the development of diseases on a larger scale [6], and thus decreases the number of hospitalised patients among the elderly [7,8]. This is particularly vital in Poland, which is expected to have the worst old-age dependency ratio among the EU countries by 2100 [9].

Health protection is one of the tasks carried out by the municipality [10]. The municipality’s tasks in the area of public health protection include, among others, providing basic healthcare, obstetrics and gynaecology services, and dentistry [11]. In order to improve the functioning and further development of PHC, the Law on Primary Health Care was enacted in 2017 [12]. Its main objective was to increase the role of PHC and to enable the coordination of its activities with other parts of the healthcare system. It was also intended to increase the role of prevention and health education. According to this act, the aims of primary healthcare are to provide healthcare for the patient and his/her family and to coordinate it in the healthcare system, to assess needs and establish the health priorities of the population covered by the care, to implement preventive actions, health promotion and the shaping of pro-health activities, and recognition, elimination, or reduction of threats to physical and mental health. The report made by the team of the Institute of Rural Medicine in Lublin in 2019 proposes seven areas of support for the development of primary healthcare in Poland, which should be included in strategic documents for the next EU financial perspective for 2021–2027. The authors mention the development of a map of the current distribution of entities at the level of individual municipalities, establishing their categorization on the basis of the number of entities providing services, the number of medical staff, and the number of patients per doctor. Then, a central fund should be created to support the development and creation of new PHC facilities in deficient municipalities, together with securing financial resources for their operation [13].

The aim of the study was to assess the spatial accessibility of statistical rural localities to primary healthcare facilities in Poland using spatial analysis and GIS methods. In addition, an attempt was made to identify PHC shortage areas as areas with the worst access to primary care with the highest population density. The research findings can serve to identify problem areas and provide support to municipal authorities and healthcare providers.

## 2. Materials and Methods

### 2.1. Study Area

**Figure 1:**
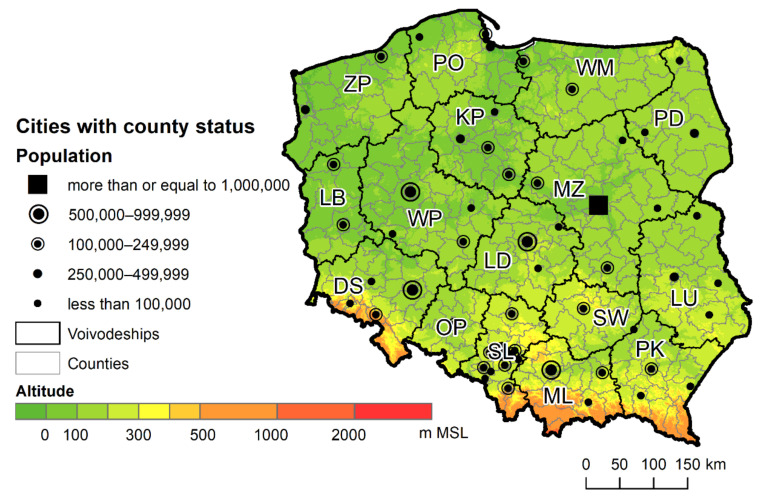
Topographic map of Poland, with the main cities and Voivodeships. Voivodeship names: DS—Lower Silesia, KP—Kuyavian-Pomeranian, LU—Lublin, LB—Lubusz, LD—Lodz, MZ—Masovian, ML—Lesser Poland, OP—Opole, PD—Podlaskie, PK—Subcarpathian, PO—Pomeranian, SL—Silesian, SW—Swietokrzyskie, WM—Warmian-Masurian, WP—Greater Poland, ZP—Western Pomeranian.

The research covered all statistical localities in Poland, a medium-sized country in Central and Eastern Europe, sixth in terms of size and fifth in terms of population in the EU [14]. Almost 35.1% of its nearly 38 million inhabitants live in rural areas [15], which is slightly above the average (27.47%) for EU countries (the OECD data did not include Cyprus). The smallest percentage of the rural population lives in the industrialised Silesian Voivodeship, with the largest percentage found in the Subcarpathian, Swietokrzyskie, Lubusz, and Lesser Poland Voivodeships [16]. The northern and central part of the country is lowland, while the southern part is occupied by uplands and mountains, including two larger mountain ranges running through the country: the Sudetes and Carpathian Mountains (Figure 1). In the northern part of Poland, especially in the Warmian–Masurian, Kuyavian–Pomeranian, and Lubusz Voivodeships, there are numerous postglacial lakes, which may have a local impact on the development of spatial accessibility. A number of watercourses run through the country, most often in the south, which may locally create linear barriers to spatial accessibility.

### 2.2. PHC Facilities

The data on the analysed primary healthcare institutions (PHC) were obtained from the National Health Fund (NFZ) via the REST network service. They contained information on all entities providing services under the agreement with the NFZ as of 2 February 2020. They also had contact details, enabling their identification in space. Geocoding of objects was carried out in two stages, using the Python GeoPy package, which is a client for common geocoding web services, such as GoogleV3, ArcGIS, and MapQuest [17]. Firstly, the free geocoder Nominatim, based on OpenStreetMap (OSM) data, was used. Since OSM creates its address layer based on, among other things, official address data from the National Register of Borders and Areas, it should be considered reliable and giving good, reproducible results. Objects whose addresses were not in the database were then geocoded using the ArcGIS module, requiring paid access to a geocoder. The points located in space, returned in WGS84 datum, either referred to specific addresses, or, if they could not be found, to points representing localities. It should, therefore, be borne in mind that for some facilities, differences between the actual location of the service and the location generated by geocoding may occur. Since the initial assessment generated no more than 5% incorrectly located points of the surveyed units, and the distances of those incorrectly inserted points were usually within a radius of several hundred metres from the actual location of the units, geocoding errors were considered statistically insignificant.

Regional branches of the NFZ, entering data into the service, coded outpatient clinics in different ways; therefore, selecting PHC facilities from among all entities providing services within the NFZ required preliminary processing of the acquired data. Ultimately, 10,285 PHC facilities were identified in the territory of Poland, having signed an agreement with the National Health Fund.

### 2.3. Localities

The basic reference unit analysed in the study were statistical localities provided by the Central Statistical Office (GUS). These are either localities distinguished for statistical purposes or groups of localities for which statistical data are compiled (https://bdl.stat.gov.pl/BDL/metadane/teryt/miejscowosc; accessed: 20 August 2021)

Out of the entire spatial dataset, rural units were identified for the purposes of analysis, and information about the population obtained from the 2011 census was assigned to them. The statistical localities were represented in the form of polygons and, for the purposes of the research, had to be reduced to a point layer. For greater accuracy of analysis, the locations of the points were based on the locations of the main settlement names from the National Register of Geographic Names. Such names were usually located in the centre of the largest settlement, which, in the absence of more detailed population data, had a similar effect as the population weighted centroid determination used by Page et al. [18]. In total, the research covered 41,603 rural statistical localities.

### 2.4. Population

Demographic data were obtained from the Central Statistical Office Local Data Bank. These data for National Censuses (NSP) are aggregated to the level of statistical localities, while in the years not covered by the censuses the smallest unit of separation are municipalities. As the last census in Poland was conducted in 2011, these data were not used directly in further analyses. However, they were used to estimate the population in the localities as of 31 December 2018. For each locality in a municipality, percentage shares of the population were calculated based on the data from NSP 2011, and then, based on the same proportions, the population was assigned to the locality based on the data from the end of 2018. This method assumes that demographic changes take place evenly in all localities in a given municipality, which is obviously a simplification. However, this approach takes into account the general changes in the population in Poland between 2011 and the end of 2018, i.e., differences in the size of localities within municipalities, which seems sufficient to obtain reliable research results.

### 2.5. Road Network

The road network data were taken from OpenStreetMap. The OpenStreetMap (OSM) project aims to create a free, worldwide digital map. Due to the large research area (the whole of Poland) and the lack of other data available for the whole country with high accuracy and timeliness, the authors decided to use the road data from the OpenStreetMap project. Owing to the volume of the file, only the classes representing the main road network system were selected (Table 1). Next, the selected dataset was subjected to a process of checking the connectivity and removing fragments of roads not connected to the network. After these activities, the total length of roads included in further analyses was 369,082.19 km.

### 2.6. Research Methods

The problem of access to healthcare facilities has a long history in geographical research, in which many additional concepts of accessibility can be found, including potential accessibility, revealed accessibility, and temporal and spatial measures of accessibility [3,6,7,8,18,19,20,21,22,23,24,25,26,27,28,29,30,31,32,33,34,35,36,37,38]. Accessibility refers to the relative ease with which one can reach different places such as workplaces, shopping centres, or a healthcare facility. McLafferty [27] describes it as the ability of people to use health services in a particular place and time.

The basic approach to accessibility research is to use indicators that are based on the relationship between the population and the number of medical facilities in a given area [27]. This is a static approach that does not represent differences in access to health services within a unit of area [34]. Another frequently used measure is the cost of travel (distance or time) to the nearest healthcare provider. It is assumed that the cost of travel to the nearest healthcare facility is a good measure, mainly for rural areas, where the choice of such facilities is very limited, which makes the closest facility the most likely choice for patients [6].

None of the above methods take into account the relationship between supply and demand [18]. Therefore, with the development of GIS techniques and tools, the 2S floating catchment area (2SFCA) method, based on the gravity model, has been used more and more often to test access to medical services [18,19,26]. This method, despite its numerous advantages, may overestimate local demand for services, leading to a disturbance in the demand–supply ratio [32]. It is based on the arbitrarily determined size of the catchment area, which can be problematic, especially when comparing the research results of different authors [28]. All locations outside the set distance or time are assigned a null value [31]. It can also conceal shortages of services provided to the population, unlike linked-based methods [33]. Some of the limitations of the 2SFCA method are solved by the E2SFCA method, which uses the distance decay function to differentiate the weights of the individual medical units depending on the distance from the facility [31,39,40]. A solution to the problem of data aggregation is attempted in the 3SFCA method proposed by Bell et al. [25], suggesting the development of the E2FCA method at larger spatial resolutions and then aggregating the data using the average to larger units (census tracts). There are also methods that take into account individual human activity and the spatial and temporal constraints related to daily, repeated duties—for example, related to an individual’s daily potential path area (DPPA) [34].

The basic measure of the assessment of spatial accessibility to the examined medical services (PHC facilities) was the shortest distance from the point indicating a given locality. This distance was calculated based on the road network model using the origin–destination cost matrix network analysis tool. The use of the shortest distance to medical services method as an accessibility measure was deliberate, despite some imperfections. Firstly, similarly to the argumentation by Yin et al. [37] referring to the average shortest distance, it was found to be an easily understandable measure for researchers and health decision makers. Secondly, as Guagliardo [6] points out and as the research by Haynes et al. [35] has shown, with longer distances between towns and thus medical facilities, this distance plays a key role in the choice of medical services by rural residents. Thirdly, country-wide modelling of travel time to medical facilities by a particular mode of transport is always subject to a certain error, which can have a significant local impact on test results.

The assessment of spatial accessibility to PHC facilities was carried out by analysing the spatial distribution of accessibility to the nearest medical services under analysis and indicating the places where it was different from the mean. The method of standard deviation was used to illustrate spatial differences in distance values, where the outliers were considered to be above and below one standard deviation from the mean.

The Theil index was used for the analysis of the spatial variability of the shortest distances from rural statistical localities to PHC facilities. Research by Yin et al. [37] has shown that it can be used to identify the variability of spatial accessibility for medical services. Since indices based on entropy allow for decomposition into intragroup and intergroup parts, the impact of the variability of accessibility of statistical localities to the surveyed medical services between counties and within counties on the Theil index was tested [41]. In its general form, the index assumes values in the range of 0–1, with a higher value meaning greater diversity of the analysed feature between spatial units, which in turn may suggest uneven access to the analysed medical services. The total value of the Theil index is calculated according to the following formula:

(1) $$T_{k}=\frac{1}{n}\overset{n}{\underset{k=1}{\sum }}\frac{y_{i}}{\bar{y}}log\left(\frac{y_{i}}{\bar{y}}\right)\;$$

where *n* is the number of units analysed and $\frac{y_{i}}{\bar{y}}$ is the ratio of the shortest distance to all PHC facilities in a given town to the average shortest distance to all PHC facilities [42].

The decomposition of the index in order to determine the intragroup and intergroup variability of the analysed availability of PHC facilities was performed in relation to counties. These formulas were used to assess intergroup and intragroup variability, respectively:

(2) $$T_{ext}\overset{m}{\underset{k=1}{\sum }}\frac{n_{k}}{n}\left(\frac{{\bar{y}}_{k}}{\bar{y}}\right)log\left(\frac{{\bar{y}}^{k}}{\bar{y}}\right)$$

(3) $$T_{int}=\overset{m}{\underset{k=1}{\sum }}\left(\frac{n_{k}}{n}\frac{{\bar{y}}_{k}}{\bar{y}}\right)T_{k}$$

where *k* means a given county, *m* is the sum of municipalities, *n_k_* is the number of localities in a county, *n* is the total number of localities, ${\bar{y}}_{k}$ is the average distance from the town to the nearest PHC in the county, and $\bar{y}$ is the average distance to the nearest PHC in total [43].

The ratio of the value of the intergroup variability to the Theil index can be interpreted as the part of the index resulting from the uneven distribution of values between counties. Accordingly, the ratio of the internal component should be understood as the part resulting from the variability of objects directly within each group, in this case within counties. A comparison of the values of intergroup Theil indexes on the map can indicate regions that are more or less diverse in spatial accessibility than others [37].

As in the paper by Yin et al. [37], to assess the spatial concentration of accessibility to PHC facilities, Moran’s I index was used to estimate the global spatial autocorrelation, while local spatial autocorrelation was used to indicate the local characteristics of the spatial accessibility under study.

Moran’s I index made it possible to determine the extent to which neighbouring or adjacent localities are characterised by similar distances to the studied PHC facilities [44]. The following formula is used to calculate the index:

(4) $$I=\frac{n\sum_{i}\sum_{j}w_{ij}\left(z_{i}-\bar{z}\right)\left(z_{j}-\bar{z}\right)}{\sum_{i}\sum_{j}w_{ij}\sum {\left(z_{i}-\bar{z}\right)}^{2}}$$

where *n* is the number of analysed localities; *w_ij_* is the weighting matrix, taking a value of 1 if the localities are adjacent to each other and 0 if not; *z_i_* is the distance to services at location *i*, *z_j_* is the distance to services at adjacent location *j*, and $\bar{z}$ is the average distance to the services analysed.

Anselin’s local Moran’s I is based on Moran’s I index and can indicate clusters of high values, low values, or outlier objects [45]. It is calculated using the formula:

(5) $$I=\frac{x_{i}-\bar{X}}{S_{i}^{2}}\overset{n}{\underset{j=1,j\neq i}{\sum }}w_{i,j}\left(x_{j}-\bar{X}\right)$$

where *x* is the distance to the nearest PHC facilities under analysis, $\bar{X}$ is the average value of the distance from all locations, *w_i,j_* are the spatial weights between the analysed location and the neighbouring location, and *S^2^* is the variance of the distance from all localities outside the analysed locality [46].

This measure designates clusters with good and poor accessibility to PHC facilities. However, this method does not take into account the demand side, i.e., the demand for the services under investigation. Therefore, these studies required the inclusion of a threshold for distance and population information that could be used to designate relatively densely populated sites where access to the above services is relatively worse.

Areas with the weakest spatial accessibility to the analysed medical services were determined using the Kernel Density function:

(6) $$f\left(x\right)=\frac{1}{nh}\overset{n}{\underset{i=1}{\sum }}K\left(\frac{x-X_{i}}{h}\right)$$

where *h* is the window width, also called the smoothing parameter, *n* is a random sample, and *x*_1_, *x*_2_, ..., *x_i_* are the points of an *n*-element random sample [47]. The *K* parameter is a function of the kernel, while *h* determines its width [48]. Many types of kernel function may be found in the literature. Following Silverman, in this research a quartic kernel function was applied [48].

The analysis of the density of clusters was determined for localities where the nearest service point was located at least 6000 m away. It was assumed that this is a distance that makes it significantly more difficult to reach such points on foot and requires the use of a means of transport. The value of the catchment radius was determined on the basis of the analysis of spatial autocorrelation, which, in the case of access to PHC facilities, reached local maximums at a distance of 7300 m.

In contrast to commonly used floating catchment area (FCA, i.e., 2FCA, E2FCA, 3FCA) or kernel density methods, this study focuses exclusively on areas with high population density and lower accessibility parameters, which may be more readable for decision makers when deciding where to locate the nearest PHC facilities in the first place in order to equalize public access to medical services. Also, this method is independent of the administrative borders used in the FCA method.

## 3. Results

**Figure 2:**
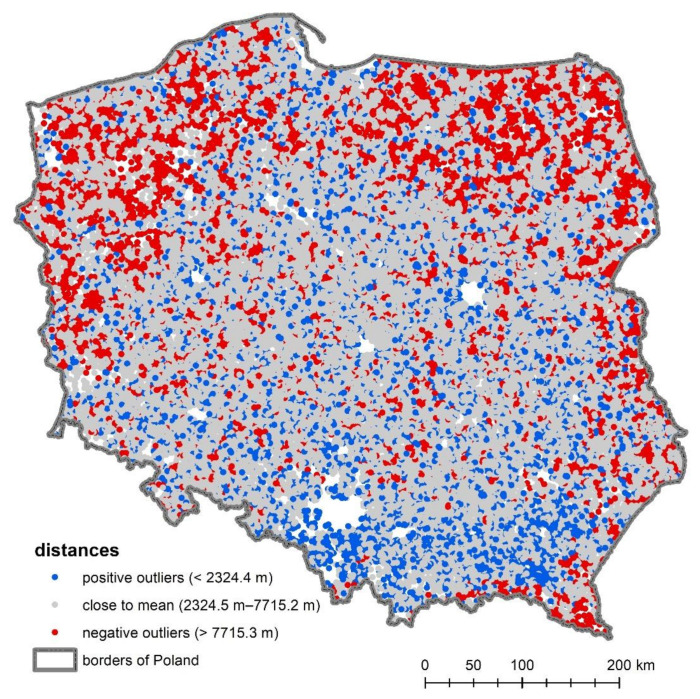
Distances to PHC facilities calculated on the road network.

In the first stage of the research, the shortest route between each of the examined rural localities and PHC facility was sought and the results were visualised on a map (Figure 2). In many cases, they showed that the nearest point shared the same address. Of PHC facilities, 51.5% are located in the closest proximity to rural statistical localities. The average distance from rural statistical localities in Poland to PHC facilities is about 5 km. There are, however, localities whose inhabitants had to travel more than 15 or 20 km to the nearest PHC facility (Figure 2), but such places constitute less than 1%.

Most of the values investigated are at a distance of one standard deviation (σ) from the mean. In the context of distance to PHC facilities, the percentage of values close to the mean, within the range of one standard deviation, is about 71.5% (Table 2).

The distance to PHC facilities varies, and its values show unequal access to medical services in different parts of the country. This phenomenon is better illustrated by a map (Figure 2), which shows villages with good, medium, and poor accessibility (standard deviation method). Larger distances to PHC facilities providing basic health services are found in the northern and eastern parts of Poland. The best access to PHC facilities (the shortest distance values) can be found mainly in rural localities in southern Poland. In the centre of the country, average values prevail, although there are areas that have worse access (Figure 2).

**Figure 3:**
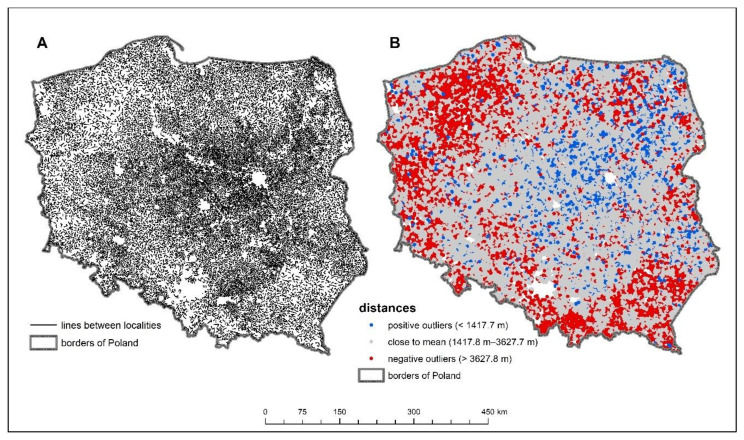
Density of the settlement network of rural statistical localities: (**A**) Connections between nearest rural statistical localities, (**B**) Localities by distance to nearest neighboring rural statistical localities.

In order to reduce errors in interpretation of this issue, the density of the settlement network of rural statistical localities was examined (Figure 3). The average distance between the surveyed villages was about 2.5 km and the maximum recorded distance was about 14.5 km. Almost 75% of all distances were within one standard deviation, which is just over 1 km. Larger distances between villages can be observed in areas near the national border, mainly in the northwestern and southeastern parts of Poland. Hence, the poor access of these areas to basic healthcare can be partly explained by the lower density of the settlement network. In the south of the country, where the distance between rural localities is large, access to PHC facilities is good, which can be explained by the large number of cities in the region. This is because cities have the largest number of establishments providing basic health services—as many as 61.4% of PHC facilities. Rural residents therefore often have to use such facilities in towns and cities, whose proximity is a significant convenience. In rural areas close to cities, especially larger ones, access to basic healthcare is better, which is visible in the south of Poland and in suburban areas of large cities. In areas where there are fewer towns and they are small, access to such services is worse, which is well illustrated by the northern part of the country (Figure 3).

The Theil index indicates small variability in the distances of individual statistical localities to PHC facilities: its value does not exceed 0.17 (Table 3). Their location in rural areas is often the result of administrative decisions at a local level. Local governments, while performing their own municipal tasks, try to at least maintain relatively good access to PHC facilities.

**Figure 4:**
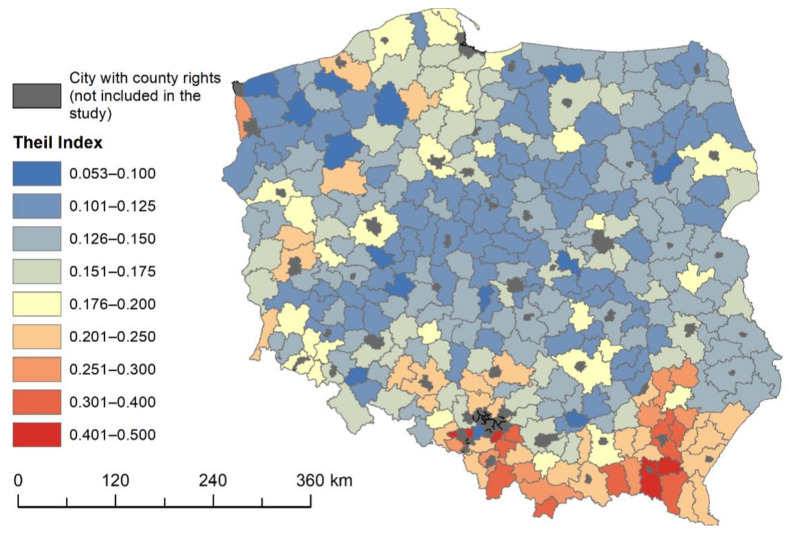
Theil Index for primary healthcare facilities.

Comparing the components of the index at the county level, it can be seen that its value is determined primarily by the intragroup diversification of accessibility to the above services. This results from the fact that, at the county level, the availability of PHC facilities is almost identical. There are, however, local variations in availability of an intragroup nature. These differences are mainly concentrated in the southern part of the country, where, depending on the county, they can even reach index values above 0.3 (Figure 4).

The maximum Theil index values are found in counties located in mountainous areas in the Carpathian arc, where the transport and settlement networks are determined by the terrain. In these areas, next to localities with good or very good access to the medical services in question, as shown by previous analyses, there are a number of villages significantly distant from them. These are counties in which local authorities should pay special attention to the need to equalize the opportunities for their residents to benefit from PHC services. From Figure 4, it can also be noted that a slightly greater diversity of accessibility to PHC facilities occurs in counties directly neighbouring the largest cities in Poland. These are areas subject to strong suburbanisation processes, where changes in spatial organisation are taking place. It is possible that access to medical services is also subject to these changes, i.e., some PHC facilities are created in new places where the strongest urbanisation processes take place. Since these changes are not evenly distributed across the county, there may be higher Theil index values in these areas.

While the Theil index shows the degree of variability of spatial accessibility, it does not answer the question of whether or not, in the case of low variability, accessibility is good or not. It also does not analyse whether there are statistically significant clusters of areas with worse or better spatial accessibility. Therefore, in the next part of the study, the degree of spatial autocorrelation and local spatial autocorrelation of the shortest distances from statistical localities to PHC facilities were analysed.

The global statistic of Moran I for the spatial accessibility of statistical localities to PHC facilities was 0.33 (Table 4). In this case, the examined results indicating low clustering of the analysed spatial accessibility to PHC facilities were statistically significant.

**Figure 5:**
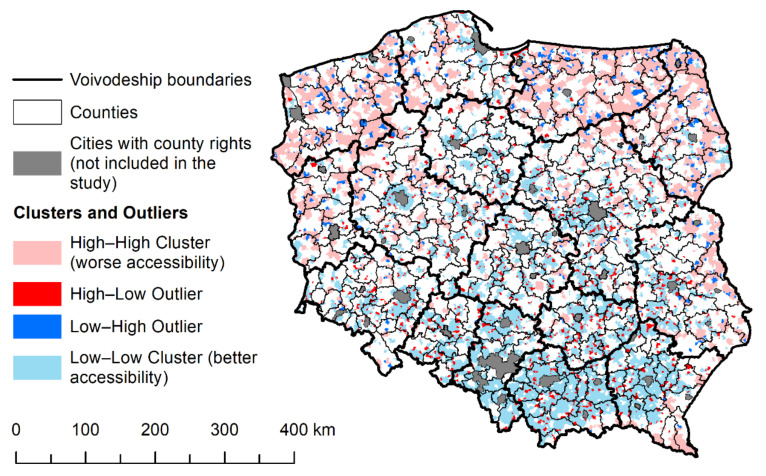
Distribution of clusters and outliers of spatial accessibility to PHC facilities in Poland.

The results of local spatial autocorrelation show great regional variation in terms of the spatial concentration of accessibility to PHC facilities (Table 4, Figure 5). Concentrations of low values, which indicate better accessibility to the services under study, were clustered in the southern central districts and in the north. On the other hand, clusters of settlements with poor accessibility were found mainly in the northern part of Poland, in the eastern part of Poland, and in peripheral (border) districts.

**Figure 6:**
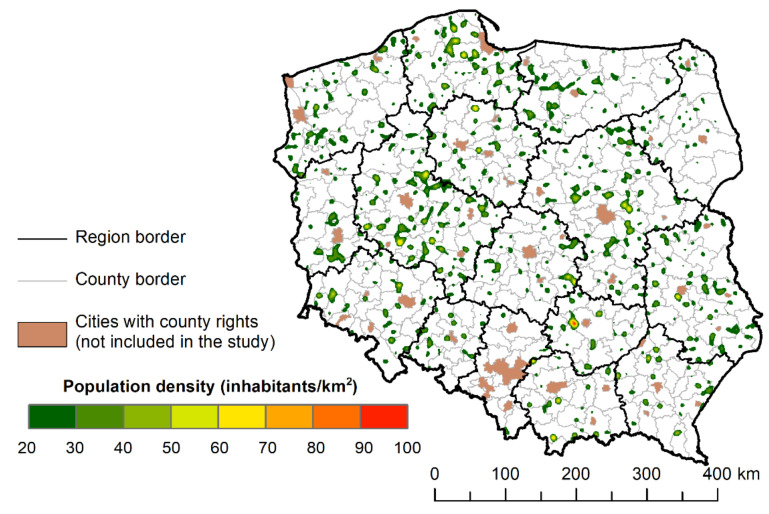
Population density in rural areas situated beyond 6 km from PHC facilities.

Areas at least 6000 m away from the nearest PHC facility were characterised by low population density and high spatial dispersion, and diversity between voivodeships. Assuming a population density limit above 20 people/km^2^, significant regional differences were observed in less accessible areas for the analysed services (Figure 6).

The largest percentage share of the area with weaker access to PHC facilities in rural areas was observed in the Masovian and Greater Poland Voivodeships. In the more strongly urbanised Silesian and in the typically rural Podlaskie Voivodeship, these areas did not cover more than 3% of the voivodeship’s area (Table 5). Referring directly to statistical localities, it has already been ascertained that poor access to healthcare facilities occurs first of all on the outskirts of voivodeships, namely, in the borderland of this voivodeship with the Kuyavian–Pomeranian Voivodeship, on the border of the Lodz voivodeships, Masovian and Swietokrzyskie Voivodeships, and in the ring surrounding Warsaw from the north, east and south, as well as in the Pomeranian Voivodeship. One wonders whether the development of settlements near the capital city has contributed to the shortage of access to the analysed medical services (Figure 6).

## 4. Discussion

Many countries, including Poland, are struggling with the problems of healthcare and the pursuit of equal opportunities in access to basic medical services, regardless of place of residence [5]. The shortage of doctors is particularly evident in rural areas [13]. This problem is very important due to the fact that good spatial accessibility to basic healthcare facilities significantly reduces the development of disease on a larger scale [6] and thus decreases the number of hospitalised patients among the elderly [7,8]. Access to healthcare is also a particularly important topic during the COVID-19 pandemic. Efficient access to a primary care physician enables faster treatment, advice, and medical care. Primary care facilities can also serve as local vaccination centres.

So far, several works on the analysis of spatial accessibility to medical services in Poland have been published [4,40,49,50,51,52], but only a few of them concern the availability of PHC facilities [51,53]. The research in relation to PHC was conducted at the municipality level [26,40,51,52]. On larger spatial scales, the accessibility of selected medical services was only studied in cities such as Cracow [53]. There have been no studies covering rural areas, so this paper is an attempt to supplement the basic knowledge in this area.

Studies of spatial accessibility to PHC in rural areas usually assume time distance, measured for car transport, but occasionally other modes of transport or pedestrian availability are considered [6,19,21,22,23,24,28,35,38,51,52,54]. The present study found that this distance varied regionally and locally. Using a smaller spatial unit in the study allowed us to identify entire areas (patches) where this accessibility was statistically significantly worse. What has not been highlighted in previous studies in Poland is the importance of physical and geographical conditions, including the topography of the terrain, on spatial accessibility to healthcare facilities. In the uplands and mountainous areas of southern Poland, significantly higher values of the Theil Index were observed, indicating a greater internal variation in the distance to the PHC. However, this variation was due to the presence of many remote localities with low populations, peripherally located in relation to the basic shape of the settlement network. Their identification and the provision of public transport in them is crucial in order to ensure equal access to healthcare, as guaranteed by the Constitution.

The cluster density method, used in the study to identify areas more densely populated and, at the same time, deprived of pedestrian access to PHC, can be equated with the determination of localities that are outside the catchment area in FCA methods. In contrast to classical FCA methods, which take values from 0 to infinity, modelling the density of population deprived of access to PHC made it possible to identify the shortage areas that suffer most from a lack of access to PHC. The authors include the demand side in their study, and as the supply side they understand the determination of shortage areas, i.e., areas where distances to PHC are greater than 6 km. The structure of shortage areas has a different spatial distribution compared to the analysis of distance to PHC.

It should be borne in mind that these results are subject to certain constraints. One such issue concerns population, which is often underestimated in suburban areas, due to lack of registration [55]. In addition, the population in 2019 was modelled and therefore may differ from the actual population of certain areas. The second constraint is related to the road network extracted from the OSM database, which does not have strict guidelines for the creation of objects. This can cause some errors or shortcomings in the course and classification of roads, especially in rural peripheral areas. There were also some errors in geocoding. For some facilities, significant differences between the actual location of the service and the location generated by geocoding occurred. It should also be noted that the poor accessibility of PHC facilities in border areas may have actually been better due to the possibility to use these services abroad.

There are a number of issues that need to be addressed in order to fully assess the accessibility of basic healthcare, including the working days and hours of facilities and pharmacies, the number of doctors and nurses working there, the number of patients they serve, and how the facilities are equipped. Unfortunately, most of these data are not publicly available in one place in Poland, and obtaining and compiling them is a difficult task, especially for such a large area. Demographic issues are closely linked to research on the accessibility of medical services, but the problem is a lack of detailed, publicly available data on the number and structure of the population in relation to individual localities.

The importance of the work is emphasised by the fact that it refers to individual localities, which makes it possible to notice the variable spatial accessibility within counties and even municipalities. This is an introduction of a new approach to the problem in the context of the research conducted so far. The validity of this type of research is confirmed by the results of the Theil index in relation to counties. In the context of variability between counties, accessibility to PHC facilities is almost the same. However, when comparing the components of the index, it can be observed that its value is determined mainly by intragroup variability, which indicates the occurrence of local access differences. The results of the local spatial autocorrelation study showed a large regional variation in the concentration of spatial accessibility.

In view of the plans related to changes in primary healthcare under the provisions of the Law on Primary Health Care of 2017 and the prospects for improving the activities of this type of services, it is necessary to identify problem areas where access to PHC and the quality of services provided are unsatisfactory. Documents are being produced informing about plans to create policies to support such areas. To designate such municipalities, we need to calculate various data and indicators, such as the number of providers of primary care services, the number of doctors and other medical staff, and the number of patients per doctor. Our research shows that adopting only such basic metrics to evaluate and identify problem areas may not be sufficient. We noted that the problem of the distance to a primary healthcare facility is an important criterion informing about accessibility, especially in rural areas.

The proposed method of identifying areas with shortages in the availability of healthcare facilities aggregates data at the level of individual localities. This approach eliminates the edge effects characteristic of indicator methods usually aggregated to the level of larger territorial units. Thus, it makes it possible to identify places in the borderland of administrative units where there may be a problem of pedestrian inaccessibility to a PHC facility. This is important because these areas are usually poorly connected, especially in terms of public transport. Their identification may be a prelude to developing common strategies with which for municipalities to improve the access of their residents to healthcare services. In addition, they point to the need for analyses to identify the needs of the residents of each locality rather than entire municipalities, due to the existing differences in access within these reference units.

The whole research process also makes it possible to identify areas characterized by a dispersed system of settlements, sparsely populated, or significantly distant from the nearest PHC. Their mapping makes it possible to develop strategies that ensure, with the lowest possible outlay on healthcare, the constitutional right to medical care for people who live there and cannot use their own means of transportation on a daily basis to reach the nearest PHC facility.

## 5. Conclusions

The main aim of the paper was to assess the spatial accessibility of primary healthcare in Poland with the use of spatial analysis and GIS methods. The aim of the research has been achieved and the results can be used in planning the PHC development strategy in rural areas in Poland. The conducted research provides an overview of the spatial accessibility in rural areas in Poland. Using the Theil Index, it was shown that variation in accessibility to PHC is intragroup in nature and that differences in access are due to local topography or location relative to major cities.

The data and research methods presented in this paper can be used to designate shortage areas and indicate places with worse spatial accessibility, which, together with other criteria, can be a basis for drawing important conclusions at the national and local levels. It was shown that the cluster density method, applied to localities described by their population and located beyond the adopted boundary, can effectively identify areas requiring special intervention in ensuring accessibility to healthcare for their residents.

In order to develop detailed analyses, it is necessary to have detailed data on the quality of services provided as well as on the actual population of a locality and its transportation capacity. Due to limited public transport in villages, research on pedestrian accessibility becomes important. The presented article is a preliminary assessment of spatial accessibility to PHC in rural areas in Poland and shows the shortage of PHC facilities in some areas. Obtaining more accurate and comprehensive data will allow us, using the presented methods, to present the full situation in Poland and may be used to monitor the situation at various time intervals in the future.

From a wider perspective, the presented research results may be used to compare the structure of spatial accessibility of rural localities to basic medical services in Central and Eastern European countries, with research carried out in other countries or regions in the world. This is particularly important because of the small number of such studies or conducting them only in small areas or relative to larger reference units, and the fact that only general indicators are taken into account in strategic and planning documents, without detailed spatial analyses. Applying the issue of access to basic healthcare in rural areas to the whole country in relation to localities is a new solution, which shows the problem in a different way.

## Figures and Tables

**Table 1:** OSM road classes with their length for Poland.

Road Class in OSM	Length in OSM for Poland after Calculation (km)
Motorway	3889.83
Trunk	5713.37
Primary	17,970.81
Secondary	31,777.92
Tertiary	90,012.95
Unclassified	72,519.37
Residential	147,197.94

**Table 2:** Main statistics on the value of distance to PHC facilities.

Mean Distance (m)	Standard Deviation σ	(−1σ; +1σ)	Maximum Value (m)	% Close to Mean	% Positive Outliers (Smaller Distances)	% Negative Outliers (Longer Distances)
5019.8	2695.4	(2324.5; 7715.2)	32,552.2	71.6	13.8	14.6

**Table 3:** Total intragroup and intergroup variability of the accessibility of PHC facilities calculated using the Theil index.

T_i_	T_ext_	T_int_
0.1668	0.0263	0.1405

**Table 4:** Spatial autocorrelation of spatial accessibility to PHC facilities.

Moran’s Index	*z*-Score	*p*-Value	% HH	% HL	% LH	% LL
0.33	209.18	0	16.48	3.86	1.60	23.96

**Table 5:** Estimated percentage of the area of voivodeship with weaker access to PHC facilities in rural areas with population density above 20 people/km^2^.

Voivodeship Name	PHC Percentage Area
Lower Silesia	3.27
Kuyavian-Pomeranian	3.34
Lublin	6.19
Lubusz	2.55
Lodz	3.08
Lesser Poland	2.00
Masovian	7.91
Opole	1.83
Subcarpathian	2.50
Podlaskie	1.67
Pomeranian	6.00
Silesian	0.45
Swietokrzyskie	2.21
Warmian-Masurian	4.31
Greater Poland	10.66
Western Pomeranian	5.75

## Data Availability

The raw data supporting the conclusions of this article are available on request from the authors.
